# Phenotypic Associations With the *HMOX1* GT(n) Repeat in European Populations

**DOI:** 10.1093/aje/kwad154

**Published:** 2023-07-06

**Authors:** Fergus Hamilton, Ruth Mitchell, Peter Ghazal, Nic Timpson

**Keywords:** ALSPAC, Avon Longitudinal Study of Parents and Children, heme oxygenase 1, *HMOX1*, phenomewide association studies, UK Biobank

## Abstract

Heme oxygenase 1 is a key enzyme in the management of heme in humans. A GT(n) repeat length in the heme oxygenase 1 gene (*HMOX1*) has been widely associated with a variety of phenotypes, including susceptibility to and outcomes in diabetes, cancer, infections, and neonatal jaundice. However, studies have generally been small and results inconsistent. In this study, we imputed the GT(n) repeat length in participants from 2 UK cohort studies (the UK Biobank study (*n* = 463,005; recruited in 2006–2010) and the Avon Longitudinal Study of Parents and Children (ALSPAC; *n* = 937; recruited in 1990–1991)), with the reliability of imputation tested in other cohorts (1000 Genomes Project, Human Genome Diversity Project, and Personal Genome Project UK). Subsequently, we measured the relationship between repeat length and previously identified associations (diabetes, chronic obstructive pulmonary disease, pneumonia, and infection-related mortality in the UK Biobank; neonatal jaundice in ALSPAC) and performed a phenomewide association study in the UK Biobank. Despite high-quality imputation (correlation between true repeat length and imputed repeat length > 0.9 in test cohorts), clinical associations were not identified in either the phenomewide association study or specific association studies. These findings were robust to definitions of repeat length and sensitivity analyses. Despite multiple smaller studies identifying associations across a variety of clinical settings, we could not replicate or identify any relevant phenotypic associations with the *HMOX1* GT(n) repeat.

## Abbreviations

ALSPAC: Avon Longitudinal Study of Parents and Children
COPD: chronic obstructive pulmonary disease
eMERGE: Electronic Medical Records and Genomics
HGDP: Human Genome Diversity Project
*HMOX1*
: heme oxygenase 1 gene
MAF: minor allele frequency
PheWAS: phenomewide association study
PGP: Personal Genome Project UK
SNP: single-nucleotide polymorphism
STR: short tandem repeat
WGS: whole-genome sequencing.

The heme oxygenase 1 gene (*HMOX1*), which encodes for the protein heme oxygenase 1, is a critical component of life. Heme oxygenase 1 is a key enzyme in the heme breakdown pathway, and it catalyzes the breakdown of heme, an iron-containing porphyrin ring, into biliverdin, ferrous iron (Fe2+), and carbon monoxide. Because heme is such a critical component of multiple biological systems, with a particularly key role in maintaining cellular stress alterations, it is suspected that this gene’s function has significant clinical manifestations (1).

In support of this hypothesis, *HMOX1* missense and nonsense mutations in humans are remarkably rare, with fewer than 10 cases reported across the literature, all of which had dramatic phenotypic associations with an increased inflammatory state, liver dysfunction, and marked iron dysregulation (2). A wide range of laboratory and animal work has identified the importance of *HMOX1*, with animal studies suggesting that up- and/or down-regulation of this gene has significant impacts on conditions as diverse as malaria (3, 4), sepsis (5), and diabetes (6).

More than 30 years ago, researchers identified a GT(n) repeat in a putative regulatory noncoding region of *HMOX1* that varies in length from about 15 copies to 40 copies. Multiple studies have identified clinical manifestations associated with the length of this repeat (last reviewed in 2004 by Exner et al. (1)). Although not all studies have identified robust associations, meta-analyses have identified associations in a variety of conditions, such as diabetes (7), chronic obstructive pulmonary disease (COPD) (8), and neonatal jaundice (9). With regard to cancer, 2 meta-analyses suggested a potential role for the *HMOX1* GT(n) repeat in cancer susceptibility among Asian populations (10, 11).

Further laboratory work has evaluated the functionality of this repeat, with most but not all studies finding that this repeat alters the inducible expression of *HMOX1* (12–18). Therefore, in this study, we aimed to identify phenotypic associations with the *HMOX1* repeat in 2 UK cohort studies—the UK Biobank study and the Avon Longitudinal Study of Parents and Children (ALSPAC)—by using a recently developed imputation reference panel including this repeat (19).

In particular, we performed 3 specific analyses: First, we searched for any phenotypic associations across the UK Biobank in a phenomewide association analysis (PheWAS); then we focused on 2 previously reported associations: infections and neonatal jaundice.

## METHODS

### UK Biobank access, genetic data, and quality control

####  

The UK Biobank is a population-based health research resource consisting of approximately 500,000 people aged 38–73 years who were recruited from across the United Kingdom between 2006 and 2010 (20). The project is particularly focused on identifying determinants of human diseases in middle-aged and older individuals. Participants provided a range of information (such as demographic characteristics, health status, lifestyle measures, cognitive testing, personality self-reports, and physical and mental health measures) via questionnaires and interviews; anthropometric measurements, blood pressure readings, and samples of blood, urine, and saliva were also taken (data available at www.ukbiobank.ac.uk). The study design, participants, and quality control methods have been described in detail previously (20, 21).

The UK Biobank study received ethical approval from the North West Multi-Centre Research Ethics Committee.

#### Genotyping and imputation

The full data release contains the cohort of participants with successfully genotyped samples (*n* = 488,377). A total of 49,979 individuals were genotyped using the Applied Biosystems UK BiLEVE Axiom Array (Thermo Fisher Scientific, Inc., Waltham, Massachusetts), and 438,398 were genotyped using the UK Biobank Axiom Array (Thermo Fisher Scientific). Preimputation quality control, phasing, and imputation are described elsewhere (20). In brief, prior to phasing, multiallelic single-nucleotide polymorphisms (SNPs) or those with minor allele frequency (MAF) ≤ 1% were removed. Phasing of genotype data was performed using a modified version of the SHAPEIT2 algorithm (22). Genotype imputation to a reference set combining the UK10K haplotype and Haplotype Reference Consortium reference panels (23) was performed using IMPUTE2 algorithms (24). The analyses presented here were restricted to autosomal variants using graded filtering with varying imputation quality for different allele frequency ranges. Therefore, rarer genetic variants were required to have a higher imputation quality information (INFO) score (INFO > 0.3 for MAF > 3%, INFO > 0.6 for MAF 1%–3%, INFO > 0.8 for MAF 0.5%–1%, and INFO > 0.9 for MAF 0.1%–0.5%), with MAF and INFO scores having been recalculated on an in-house–derived “European” subset (21).

#### Data quality control

Individuals with mismatched sex data (derived by comparing genetic sex with reported sex) or sex chromosome aneuploidy were excluded from the analysis (*n* = 814). Individuals who were outliers in heterozygosity and missing rates (*n* = 968) were also excluded.

We restricted the sample to persons of “European” ancestry as defined by an in-house *k*-means cluster analysis performed using the first 4 principal components provided by the UK Biobank in the statistical software environment R (R Foundation for Statistical Computing, Vienna, Austria). The current study included the largest cluster from this analysis (*n* = 464,708).

### Avon Longitudinal Study of Parents and Children

ALSPAC is a longitudinal birth cohort study in which 14,541 pregnant women resident in Avon, United Kingdom, with expected dates of delivery between April 1, 1991, and December 3, 1992, were recruited. Of these initial pregnancies, there were a total of 14,676 fetuses, resulting in 14,062 live births and 13,988 children who were alive at 1 year of age. The children and their mothers have been followed up through postal questionnaires and at clinics (25, 26). The study’s website contains details on all the data that are available through a fully searchable data dictionary and variable search tool (27). Ethical approval for the study was obtained from the ALSPAC Ethics and Law Committee and local research ethics committees.

ALSPAC children were genotyped using the Illumina HumanHap550 QuadChip (Illumina, Inc., San Diego, California) genotyping platform, and genotypes were called with Illumina GenomeStudio (Illumina, Inc.). Imputation was performed using the Michigan Imputation Server (University of Michigan, Ann Arbor, Michigan) with phasing via Shape-IT (28). SNP-level quality control removed variants with more than 5% missingness or *P* values for Hardy-Weinberg equilibrium smaller than 1 × 10^−6^. Participant-level quality control removed variants with uncertain X chromosome heterozygosity, extreme autosomal heterozygosity, or more than 5% overall missingness. Next, multidimensional scaling of genomewide data was performed including reference data from HapMap populations (29). Samples which clustered outside the CEU population (Northern Europeans from Utah) were removed. We limited SNPs to those with an INFO score greater than 0.8.

### Other cohorts

For testing the robustness of the imputation, whole-genome and SNP array data were downloaded from 3 additional data sets: the Human Genome Diversity Project (HGDP) (30), the Personal Genome Project UK (PGP) (31), and the 1000 Genomes Project (32). All of these data sets are freely available without prior ethical approval.

### Imputation approach

Although we briefly describe the imputation approach here, a fuller description of the imputation approach and metrics is provided in the Web Appendix (available at https://doi.org/10.1093/aje/kwad154).

An imputation reference panel for short tandem repeats has previously been developed for use with SNP array data (19). This was developed using linked SNP array/whole-genome sequencing (WGS) data from 2 cohorts (Simons Simplex Collection and 1000 Genomes). The HIPStR tool (33) was used to call repeat length from WGS data, and then a reference panel was developed for use in downstream imputation.

For this study, genomic data for a 2-Mb region on either side of *HMOX1* were extracted from the above data sets and lifted over to the GRCh37 reference, if not on it already. Alleles were conformed using the conform-gt program (34) to ensure they were the same as the reference genome, and then Beagle 5.2 (35) was used with standard settings with a window of 2 Mb to impute the *HMOX1* short tandem repeat (STR), using the previously developed reference panel (19). Only high-quality SNPs (INFO > 0.8) were included.

#### Allelic calling and genotyping

Because the repeat length varies in a linear fashion, the main analysis was conducted using the sum of both allelic lengths as a linear predictor of phenotypes. The length was calculated from the number of full GT repeats. We chose to include the repeat as a linear approach (as opposed to looking at each individual’s haplotype) because 1) prior associations at this repeat have always been linear and 2) nearly all previously published STR associations have been linear (1, 36).

For sensitivity analyses, various allelic definitions were tested, including an allelic model (where each allele was analyzed separately) and a model using repeat length cutoffs to define alleles. There is significant variation in the literature around what defines an appropriate cutoff, with both binary (short vs. long) and ternary (short, medium, and long) models, with a wide variety of cutoffs used across prior studies. Given this variation, and with no empirical evidence to support previous definitions, we chose to simply quartile the alleles and include them in the model (37).

#### Imputation quality control

We tested the imputation quality in 4 discrete data sets (UK Biobank, PGP, HGDP, and 1000 Genomes) with combined high-quality (>20× coverage) WGS data and SNP array data available for the same sample. For the WGS data, the locus was called directly from the binary alignment map (BAM) files using HIPStR version 0.6.2 with standard settings (38). Subsequently, standard filtration to remove low-quality calls was performed as per HIPStR standard settings, and only samples with a posterior probability of the correct genotype greater than 0.9 were kept. Full details on the imputation quality control and testing are given in the Web Appendix (Web Tables 1–3, Web Figures 1–4).

Quality control was not performed on the imputed data, since initial experimentation identification found that the posterior probability of genotype calling was not predictive of accuracy (Web Appendix).

The quality of imputation using the haplotype reference panel has previously been reported to be good at this locus, particularly in European populations (39). Three imputation metrics were calculated: the concordance (the fraction that exactly matched the allele length across both data sets); Pearson’s correlation coefficient (*R*), calculated on the sum across both alleles; and the fraction concordant to within 2 repeats, since most previous analyses have suggested a linear relationship between STR length and outcomes (36). As a final test of imputation, we imputed the AC promoter repeat in the protein requiring fifty three 1 homolog gene (*RFT1*) in the UK Biobank. This repeat has been previously associated with height in the Electronic Medical Records and Genomics (eMERGE) cohort (36).

### UK Biobank PheWAS

Using the above genotypes for *HMOX1*, we performed a PheWAS across the UK Biobank participants for a wide variety of traits using established software (PHESANT) (40). Traits analyzed included algorithmically defined health outcomes extracted from electronic health record data (e.g., diagnoses), anthropometric traits, biological sample traits (e.g., protein levels), health questionnaires, and mortality data. In total, 7,901 traits were included. Full details on the pipeline are available in the original publication (40), but briefly, for linear traits, linear regression was used; for binary traits, logistic regression was performed; for ordered categorical traits, ordinal logistic regression was used; and for unordered traits, multinomial regression was used. The analysis was carried out using age, sex, genetic chip, and the first 10 principal components as covariates. A complete-case analysis was performed for each trait (i.e., no imputation).

As a sensitivity analysis, the analysis was conducted without any covariates and using repeat length split into equal groups to explore any nonlinear effects. Previous studies have used a variety of cutoffs, with no clear evidence to support any particular allelic definition. Therefore, we simply quartiled the exposure and reran the analysis.

### UK Biobank infection-specific analyses

Because *HMOX1* is a stress response gene, it is plausible that any genetic variation has an impact only in the presence of cellular stress; so, in a cohort type analysis, no signal of variation would be identified, despite a signal during cellular stress. In particular, infection has been suggested as a particular cellular stressor, and severe infection is known to highly up-regulate *HMOX1*, while knockout models of *HMOX1* in animals show markedly worse outcomes with infection.

**Table 1:** Overall Performance of a Recently Developed Imputation Reference Panel Including the *HMOX1* GT(n) Repeat Across 4 Test Data Sets^a^

**Study**	**Pearson’s *R***	**Concordance**		**No. of Cases**
		**Exact**	**Within 2 Repeats**	
Overall	0.907	0.455	0.837	2,916
1000 Genomes Project	0.914	0.460	0.835	2,133
Human Genetic Diversity Project	0.876	0.426	0.848	656
Personal Genome Project	0.900	0.469	0.771	83
UK Biobank	0.936	0.590	0.932	44

^a^ Data were obtained from the 1000 Genomes Project, the Human Genetic Diversity Project, the Personal Genome Project, and the UK Biobank.

Therefore, we extracted cases of infection from the UK Biobank and used Cox regression to estimate hazards for 28-day mortality, with *HMOX1* allele length as a predictor. Definitions of infection were developed by an infectious disease specialist (F.H.) using *International Classification of Diseases, Tenth Revision*, coding, and cases were extracted from the UK Biobank–linked electronic health record data (41). Codes for each infection are available in Web Table 4. Cox regression was performed using the “survival” package in R (42), both unadjusted and adjusted for age and sex.

Because patients often had multiple episodes of infection, only the latest infection was taken for this analysis, to avoid immortal time bias.

### ALSPAC neonatal jaundice

Because *HMOX1* promoter variation has been shown to influence rates of neonatal hyperbilirubinemia, data on bilirubin levels around birth and the date of sampling were extracted for 937 ALSPAC participants. Definitions of neonatal jaundice vary between governing bodies, with both the time since birth (in hours) and the level of the bilirubin result influencing case definitions. For ALSPAC participants, we had the highest recorded bilirubin result, and the day after birth on which this result had occurred.

Therefore, we took 2 approaches. First, we used the National Institute for Health and Care Excellence nomogram for bilirubin levels to generate cases of neonatal hyperbilirubinemia, and then we performed a case-control analysis with the *HMOX1* promoter genotype as a predictor of case status (43). Because numbers of neonatal jaundice cases were expected to be low in this birth cohort, we subsequently developed a *z* score for each participant for the given day on which the sample was taken, so each participant with a result had a *z* score for the bilirubin result for a given day. The repeat length of the *HMOX1*’s STR was then tested as a predictor of *z* score, in a linear fashion.

## RESULTS

### Imputation quality

The quality of the imputation was tested across 4 discrete data sets (1000 Genomes, HGDP, PGP, and UK Biobank; Table 1, Web Figure 5), all of which had both SNP array data and high-quality WGS data. In particular, we compared Pearson’s *R* values for total repeat length across both alleles and the exact concordance (both alleles exactly correct).

Overall, the correlation between the summed lengths of both alleles was excellent, with a Pearson’s *R* of 0.91 in the whole cohort. However, concordance—where both alleles were correctly called to the exact repeat—was much weaker, with a summary concordance of 0.45. Importantly, however, more than 80% of all called lengths were accurate to within 2 repeats, and more than 90% across the UK Biobank, our main cohort. The median absolute difference in repeat lengths was 1 (interquartile range, 0–2), and there was no evidence of systemic positive or negative bias in imputing (mean difference between imputed and true repeat length = −0.0013 repeats). Web Figure 6 shows the median absolute difference in total repeat length across the 4 cohorts.

Because there might have been evidence that imputation quality differed between populations, given the different reference haplotypes across populations, we calculated the imputation quality for populations, where this was reported in HGDP and 1000 Genomes (Web Appendix). Since both the PGP and the UK Biobank are entirely based in the United Kingdom, this analysis was not undertaken for those 2 cohorts. Overall, there was some evidence that imputation quality differed between populations, with slightly worse performance in the HGDP superpopulations Africa (*n* = 50, *r* = 0.73), East Asia (*n* = 158, *r* = 0.84) and Oceania (*n* = 21). Importantly for this study, there was high correlation between the true repeat length and the imputed repeat length in populations with British ancestry, indicating that the imputation was of high quality, with correlations above 0.9 for the UK Biobank, the PGP, and GBR (British from England and Scotland) populations in 1000 Genomes and HGDP.

As a final test of imputation in the UK Biobank, we imputed an AC repeat in *RFT1* as a positive control, since it had a known association with height in prior analyses (36). In the study by Fotsing et al. (44), this repeat was imputed in the eMERGE cohort, where a robust positive association with height was identified (*P* = 0.00328; β = 0.010; *n* = 6,393, for each AC repeat copy).

**Table 2:** Previously Identified Associations Between Specific Health Conditions and the *HMOX1* Promoter, UK Biobank, United Kingdom, 2006–2010

**Diagnosis**	**No. of Cases**	**β**	**95% CI**	***P* Value**
Diabetes mellitus				
Type 2	35,917	0.01	0, 0.02	0.088
Type 1	4,407	0.01	−0.02, 0.04	0.417
COPD	20,539	−0.01	−0.02, 0.01	0.278
Pneumonia	31,893	0.00	−0.01, 0.01	0.628

Abbreviations: CI, confidence interval; COPD, chronic obstructive pulmonary disease; *HMOX1*, heme oxygenase 1 gene.

We replicated this analysis in the UK Biobank, with 441,832 participants who had both genotype and standing height data available for analysis. Reassuringly, we found almost the exact same effect size (β = 0.011, *P* < 1 × 10^−16^; Web Figure 7), showing a small increase in height with increasing repeat length.

In summary, imputation at the *HMOX1* locus in the UK Biobank was reliable (Pearson’s *R* > 0.9 in British populations), and we were able to replicate other STR-phenotype associations from other cohorts.

### UK Biobank imputation metrics

After quality control and filtering (described above and in Mitchell et al. (21)), 463,005 individuals were included in the imputation pipeline. Because we performed no filtering postimputation (see Web Appendix for reasoning), we called *HMOX1* repeat length on 463,005 samples. Web Figure 8 shows the logged distribution of the allele lengths for 1) individual alleles and 2) summed repeat length across both alleles. We performed a χ^2^ test to compare proportions of homozygotes and heterozygotes at each allele length; the test showed that Hardy-Weinberg equilibrium was not exceeded (*P* = 0.265).

As has been shown previously, the *HMOX1* polymorphism has a trimodal distribution, with major peaks at 25, 32, and 39 repeat lengths (1), although in this analysis the longer repeat length is much rarer, with 32 being by far the most common repeat length.

### PheWAS analysis

For the main PheWAS analysis, we tested 7,901 variables, using previously described software (PHESANT), and taking *HMOX1* repeat length as a linear variable (40). The quantile-quantile plot is shown in Web Figure 9; the figure shows limited deviation only at the extreme end. No associations met either a Bonferroni-adjusted or false-discovery-rate–corrected *P* value.

In our sensitivity analyses, we performed the PheWAS without adjustment for principal components or UK Biobank assessment center and quartiled the exposure, to ensure that our definition of the exposure (summed repeat length) did not alter the results (Web Figure 10). For the latter 2 models, we had results similar to those of the main model, while in the former model (without adjustment), we identified associations with some sociodemographic variables (e.g., place of birth, month of visiting the assessment center) but did not identify any clinically relevant associations.

### Specific associations

Because pneumonia, COPD, and diabetes have all previously been identified as having an association with the *HMOX1* promoter repeat, we extracted data on these clinical variables from the PheWAS analysis. None showed evidence of an association with *HMOX1* repeat length (Table 2).

### Mortality within infection analyses

Because the impact of *HMOX1* repeat polymorphisms might only be present in specific environmental associations such as those involving severe cellular stress, and because there is some evidence that *HMOX1* polymorphisms alter survival from severe infection (14, 45), we undertook a survival analysis for 11 coded infections within the UK Biobank (Table 3).

**Table 3:** Infections Exhibiting an Association Between the *HMOX1* Promoter and 28-Day Mortality, UK Biobank, United Kingdom, 2006–2010

**Diagnosis**	**No. of Cases**	**28-Day Mortality, %**
LRTI	61,864	1.66
URTI	51,143	0.07
SSTI	49,369	0.70
Pneumonia	35,299	13.63
Gastroenteritis	32,389	2.07
UTI	17,488	0.14
Sepsis	14,841	16.78
Appendicitis	10,265	0.13
Osteomyelitis	7,793	1.03
Cholecystitis	5,974	1.00
Endocarditis	1,411	14.17

Abbreviations: *HMOX1*, heme oxygenase 1 gene; LRTI, lower respiratory tract infection; SSTI, skin and soft tissue infection; URTI, upper respiratory tract infection; UTI, urinary tract infection.

Infections with a low 28-day mortality were excluded from the analysis, and only 3 infections were taken forward for formal modeling (endocarditis, pneumonia, and sepsis). In both unadjusted and adjusted (for age and sex) models, there was no association between *HMOX1* repeat length and hazard of 28-day death (Table 4).

**Table 4:** Hazard Ratios for 28-Day Mortality From Specific Health Conditions With Increasing *HMOX1* GT(n) Repeat Length, UK Biobank, United Kingdom, 2006–2010^a^

**Diagnosis**	**HR**	**95% CI**	***P* Value**
Endocarditis	1.004	0.977, 1.030	0.76
Unadjusted			
Adjusted	1.004	0.977, 1.030	0.75
Pneumonia	1.003	0.997, 1.010	0.29
Unadjusted			
Adjusted	1.003	0.997, 1.010	0.36
Sepsis	1.001	0.993, 1.010	0.87
Unadjusted			
Adjusted	1.001	0.993, 1.010	0.83

Abbreviations: CI, confidence interval; HR, hazard ratio; *HMOX1*, heme oxygenase 1 gene.

^a^ Adjusted results were adjusted for age and sex.

### Neonatal jaundice

Previous systematic reviews have identified an association between the *HMOX1* promoter and neonatal jaundice (9). In ALSPAC, a proportion of children had (clinically driven) bilirubin testing during their postnatal care. In total, 937 children had a bilirubin level recorded within 14 days of birth and were successfully genotyped for the *HMOX1* promoter repeat, using the same pipeline as above. Web Figure 11 shows the sample distribution, with most samples taken in the first 72 hours of life.

Using the National Institute for Health and Care Excellence definition of jaundice requiring phototherapy, we calculated the number of cases of neonatal jaundice across our sample. ALSPAC only reports the date of the maximal bilirubin level, so each child had a single result attached to a single day (43).

In total, 47 cases of hyperbilirubinemia were identified, with the vast majority identified on day 2 (7 cases), 3 (19 cases) or 4 (17 cases) of life. In a logistic regression model, there was no association between total repeat length and neonatal jaundice (odds ratio = 1.01, 95% confidence interval: 0.88, 1.14) and no difference in average repeat length between cases and controls (mean repeats 27.3 for each group).

Given the low number of cases, we next performed an analysis using a *z* score for each individual result within each day (e.g., each day was treated individually, given the known association between postnatal age and bilirubin level). An association of the *z* scores with repeat length was tested by linear regression (Web Figure 12). Again, we failed to identify an association between *z* score and *HMOX1* repeat length (β < 0.01, *P* = 0.97).

In summary, we could not identify any association between the repeat length polymorphism and either clinical jaundice or an increase in bilirubin levels in neonates, contrary to previous reports.

## DISCUSSION

In this study, we imputed the *HMOX1* repeat polymorphism in 2 well-described cohorts, the UK Biobank and ALSPAC. Imputation accuracy was assessed using external cohorts with high-quality WGS data and was generally found to be high (Pearson’s *R* was approximately 0.9 for imputed repeat length), although concordance with the exact length was lower (around 45%). Using these imputed genotypes, we performed a PheWAS (in the UK Biobank) and tested 4 specific associations from the literature (pneumonia, COPD, diabetes, and neonatal jaundice). Notably, we found that the *HMOX1* repeat length was not associated with survival from 3 important infections (pneumonia, sepsis, and endocarditis). Further, while the authors of a meta-analysis of smaller studies (7–9) reported associations with COPD, diabetes, and neonatal jaundice, we found no associations.

### Strengths

The major strength of this study was the size of the cohort (for the UK Biobank especially), with well-characterized clinical metadata. We performed extensive external testing of the imputation approach in 4 differing cohorts and found that the imputation accuracy was reliable, particularly in European populations, lending weight to our results. Additionally, we tested other identified associations with a repeat promoter in *RTF1* and were able to replicate others’ results, suggesting our imputation approach was robust.

### Weaknesses

There were 3 main weaknesses of this study. The first is that genotypes were imputed, not directly called. However, this is true for many SNPs in most genetic studies, and we confirmed the reliability of this imputation in 4 separate data sets, while other published data support the reliability of the imputation (19). Although the imputed repeat length was highly correlated with true repeat length, it was much less reliable at calling the exact allele length (approximately 45% correct). This was partly due to a large number of potential alleles (64 potential repeat lengths), with a smaller number of common alleles, making imputation technically difficult. Although this technical limitation should be recognized, it is important to note that all prior associations with this repeat have been with repeat length, with no data that we are aware of suggesting the effect is related to a particular allele rather than the total length of the repeat (1, 7–10, 14, 16, 45, 46).

Second, a genomewide analysis of 2,060 expression short tandem repeats found linear associations to be the most common association between STRs and gene expression—a finding also identified in other studies of human STRs (36, 47–49). We therefore suggest that the lack of association with repeat length and multiple phenotypic outcomes in this analysis is valid.

Finally, in common with all cohort analyses, we were limited by the sampling frame of the study population, which does not represent the wider UK population, and were limited in the outcomes that were recorded, which were largely algorithmically defined from linked electronic health data or obtained via self-report. However, the quality of linked electronic data in the UK Biobank is excellent, and UK Biobank data have been used widely in PheWAS analyses (50).

### Comparison with previous literature

It is worth exploring the discrepancy between our results and others, particularly in neonatal jaundice and in diabetes, where the strongest prior evidence for an association has been identified. There are multiple possible explanations which are not all mutually exclusive. The simplest explanation is that the null result is correct. The previous studies were all much smaller, with most studies comprising under 1,000 patients, with potential biases due to selection of controls, definition of alleles, or the genotyping process, and the evidence for any effect is only present in meta-analyses. It is well established that genetic effects are generally small, and this may simply reflect the common phenomenon of larger studies' identifying null results while smaller, earlier studies suggest an association.

However, other possible explanations are plausible. First, nearly all previous studies have classified alleles into short, medium, or long, based on a variety of classification systems. In a previous review of *HMOX1* polymorphisms and infection, we showed that this classification was inconsistent and that misclassification creates a significant risk of bias (45). For example, in the previous meta-analysis on type 2 diabetes, one study was excluded because the allelic definition was too different from the other studies; the authors defined short alleles as under 25–27 repeats and long alleles as 25–27 repeats (7). However, given that 1) the trimodal distribution peaks at 27 repeats, 2) definitions of repeat length vary, and 3) there are generally small errors and variability in genotyping by fragment length polymorphism, these definitions are highly questionable and are at significant risk of biasing studies. It may be that through selection of allelic definitions, artificial associations were identified.

Second, our study was performed entirely in a European population, whereas many of the previous positive studies have been performed in other populations, particularly in East Asia and Africa. Because the *HMOX1* repeat association may be not causal but simply coincident with a local SNP haplotype, it may be that there is an association between certain SNPs in the *HMOX1* promoter and outcomes that are simply not present at a sufficiently high frequency in a European population. Recent studies have suggested that variable number tandem repeats have differing effects on differing haplotypic backgrounds, although this has not been shown for *HMOX1* (51). This underscores the importance of further genetic research in non-European populations to increase our understanding of effects of genetic variation on complex traits.

Third, although we tested our imputation approach extensively and are confident of its accuracy to within 2 repeats, it may be that the imputation method is biased with respect to important outcomes. This may be particularly relevant if, for example, a set of SNPs that are causal for the outcomes and are usually coincident with an increased repeat length are systematically imputed incorrectly. Although this association is possible, it would have been extremely unlikely here; given the number of cases in the UK Biobank, we would have had ample statistical power to detect even attenuated exposures, if the “true” effect size was similar to that in previous studies.

Fourth, it is possible that the repeat length has insufficient functional impact on the regulation of *HMOX1* expression. Further experimental studies are required to rigorously assess the functional role, if any, of the repeat length directing or altering the expression of *HMOX1.* Our findings in this report challenge the accepted view that the repeat length affects the biological role of *HMOX1*.

Finally, although we were able to rule out associations between *HMOX1* repeat length and incident conditions such as diabetes, we could not easily explore the association between an inducible stress response gene and relevant outcomes (e.g., infection outcomes) in a biobank analysis. In previous in vitro analyses, the baseline expression of *HMOX1* was unchanged with differing repeat lengths. However, inducible expression is highly varied in some (but not all) in vitro work (12, 14, 16, 52). This suggests that the effect of *HMOX1* repeat polymorphisms may only be present under certain phenotypic conditions (e.g., cellular stress secondary to inflammation or infection). Even given the size of the UK Biobank cohort, it is difficult to explore these potential interactions.

For example, if *HMOX1* repeat length only has phenotypic implications in the critically unwell patients with infection, we are limited to those patients in the UK Biobank who develop critical infection—an extremely heterogenous condition—while the only outcome measure we can reliably record is mortality.

### Summary

In summary, we did not identify any associations between the *HMOX1* repeat length polymorphism and any clinical outcomes in 2 well-characterized European cohorts. Reconciling this work with previous work is difficult, suggesting either no association or a more complex gene-environment interaction for the *HMOX1* repeat.

### Conclusion

In this study, the *HMOX1* GT(n) repeat was not associated with any phenotypes in the UK Biobank. Previous associations with diabetes, COPD, and pneumonia were not replicated. In a separate analysis, *HMOX1* GT(n) repeat length was also not associated with neonatal jaundice in a longitudinal cohort study, failing to replicate previous findings.

## Supplementary Material

Web_Material_kwad154
