# Supplementary material for: Phenotypic Associations With the HMOX1 GT(n) Repeat in European Populations
Source: Am J Epidemiol. 2023 Jul 6;193(5):718–26. doi: 10.1093/aje/kwad154 (PMC11074708; doi:10.1093/aje/kwad154)
Supplement: Web_Material_kwad154 [file web_material_kwad154.zip › kwad154 Hamilton Web Material.pdf]

WEB MATERIAL

Phenotypic Associations With the *HMOX1* GT(n) Repeat in European Populations

Fergus Hamilton, Ruth Mitchell, Peter Ghazal, and Nic Timpson

<u>Contents</u>

Web Appendix

Web Table 1

Web Table 2

Web Figure 1

Web Figure 2

Web Figure 3

Web Table 3

Web Figure 4

Web Table 4

Web Figure 5

Web Figure 6

Web Figure 7

Web Figure 8

Web Figure 9

Web Figure 10 (see separate file)

Web Figure 11

Web Figure 12

References

WEB APPENDIX

Imputation Testing and Quality Control

Background and WGS genotype calling

The accuracy of the imputation of the HMOX1 STR from SNP data is critical to downstream analysis. Previously, Saini and colleagues had developed a haplotype reference panel for a wide range of STR's and tested it on the 1000 Genomes project and Simon's Simplex Collection (SSC) .[1] As 1000 Genomes and SSC have ~ 30X WGS data, the STR can be called reliably, and the quality of the imputation compared with the SNP called imputation. Using this data, they reported reasonable quality imputation in European populations and in SSC but weaker performance in other populations.

The summary Pearson's correlation in European populations was 0.97 and in SSC (largely European) the corresponding figure was 0.93. To confirm these figures, we replicated the analysis in four other cohorts with linked WGS data where the microsatellite length can be accurately called – The Human Genome Diversity Project (HGDP, n = 826),[2] UK Biobank (UKB, n = 44), the UK Personal Genome Project (PGP, n = 88), and the 1000 Genomes project (1000G, n = 2318).

For HGDP, 1000G, and PGP, genetic data is freely available and the quality control on data is described in the relevant publication. For UKB, we utilised genetic data which had undergone in house quality control as described here.[3] At the time of publication, only a pilot sample of UKB participants had undergone whole genome sequencing, hence the low numbers of included participants (n = 44).

For HGDP, UKB, and PGP, we downloaded BAM files from the respective cohorts, and used HipSTR v0.62 with standard settings to measure the STR repeat length. This uses a hidden Markov model to genotype and phase short tandem repeats while taking account of sequencing artifact. We then performed the standard recommended filtering of genotype calls, filtering on the posterior probability (>0.9), and other technical aspects of the STR calling process as described here.

For 1000G, we contacted the authors of Saini et al, who kindly provided their WGS STR genotype calls directly.[1]

HMOX1 STR imputation

Subsequently, we then performed imputation using the haplotype reference panel on SNP array data from each cohort, using Beagle 4.2 with standard settings. Each cohort was imputed separately. Table S2 describes the number of included cases for comparison. We imputed across 1mb either side of the HMOX1 repeat, after initial experimentation and published data found no beneficial effect of larger imputation windows.[1]

Alleles were called based on the most probable allele as reported by Beagle, with no filtering due to posterior genotype probability (discussed in the section below).

Web Table 1. Number of samples with both SNP and array data available

Study	Total	Imputed (SNP)	WGS Accurately	Both Available
1000G	2318	2318	2133	2133
HGDP	826	800	676	656
PGP	88	87	83	83
UKB	44	44	44	44

<u>Comparison of WGS vs imputed repeated length</u>

For our initial analysis, we can simply compare the imputed repeat length (total) vs the WGS called repeat length (total). As described in detail in other sections, as HipSTR calling cannot phase a genotype (i.e. know which strand a read has come from), we have to assess the accuracy of the imputation at the genotype level, not the allelic level. All lengths were measured as the number of GT repeats.

We chose three metrics: Pearson's correlation, which was simply calculated across the total allele length, exact concordance (total repeat length exactly matches the imputed length), and within two repeats (to identify the proportion of total lengths that were close to the exact length).

Web Table 2. Metrics of imputation performance across each of the study cohorts

Study	Pearson's Correlation	Concordance (Exact)	Within 2 Repeats	No.
Overall	0.907	0.455	0.837	2916
1000G	0.914	0.460	0.835	2133
HGDP	0.876	0.426	0.848	656
PGP	0.900	0.469	0.771	83
UKB	0.936	0.590	0.932	44

As can be seen, across all four cohorts, the Pearson's correlation is > 0.85, and generally > 0.9, suggesting a very strong correlation between imputed and directly genotyped repeat length, backed up by the fraction of samples that have a total length within two repeats being > 0.80 in all but one cohort. However, the actual concordance - the number of repeat lengths that are exactly accurate, is lower. This is to be expected, as minor differences in repeat length in either allele will alter this. This can be seen visually in Web Figure 1 (next page), which plots all four studies on top of each other.

Web Figure 1. Imputation quality across each superpopulation in HGDP.

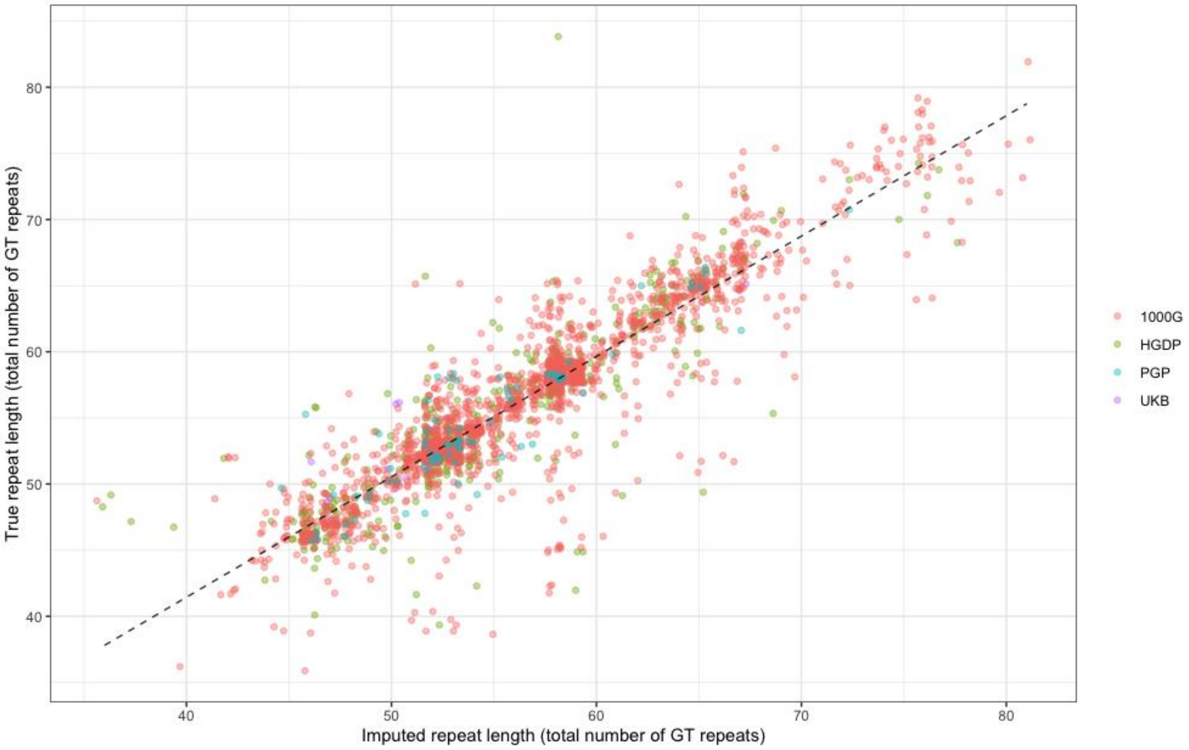

<u>Geographic variation</u>

It is likely that the imputation reference panel will perform differently in different populations. We therefore extracted superpopulation and population for the two cohorts where this was available (HGDP and 1000G).

In Web Figure 1, the imputation quality is plotted for each super population in HGDP against the number of participants. For all superpopulations except Oceania, the Pearson's correlation was >0.7, but with evidence of varying imputation performance across each superpopulation.

In Web Figure 2 (next page), we show the imputation performance across each population (n>10) in both 1000G and HGDP, again showing a large variation in imputation performance across sub populations. Web Table 3 shows the imputation performance for all three metrics within each population.

Web Figure 2. Imputation performance across each population (n>10) in both 1000G and HGDP.

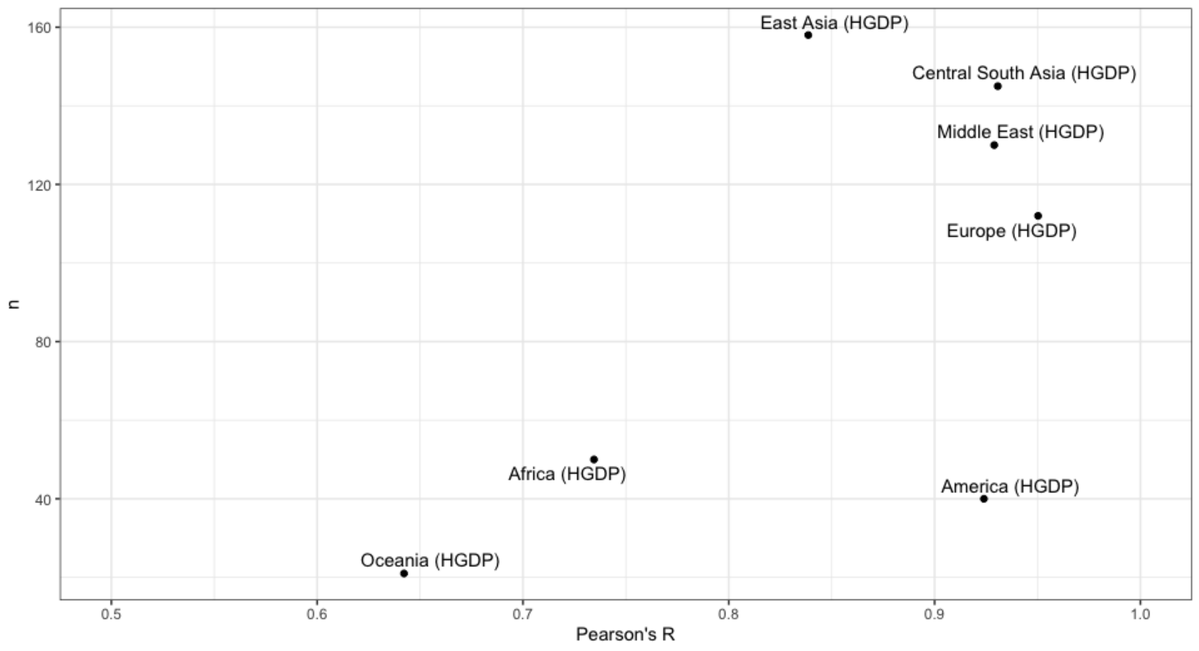

Web Figure 3. Imputation performance across each population in HGDP and 1000G.

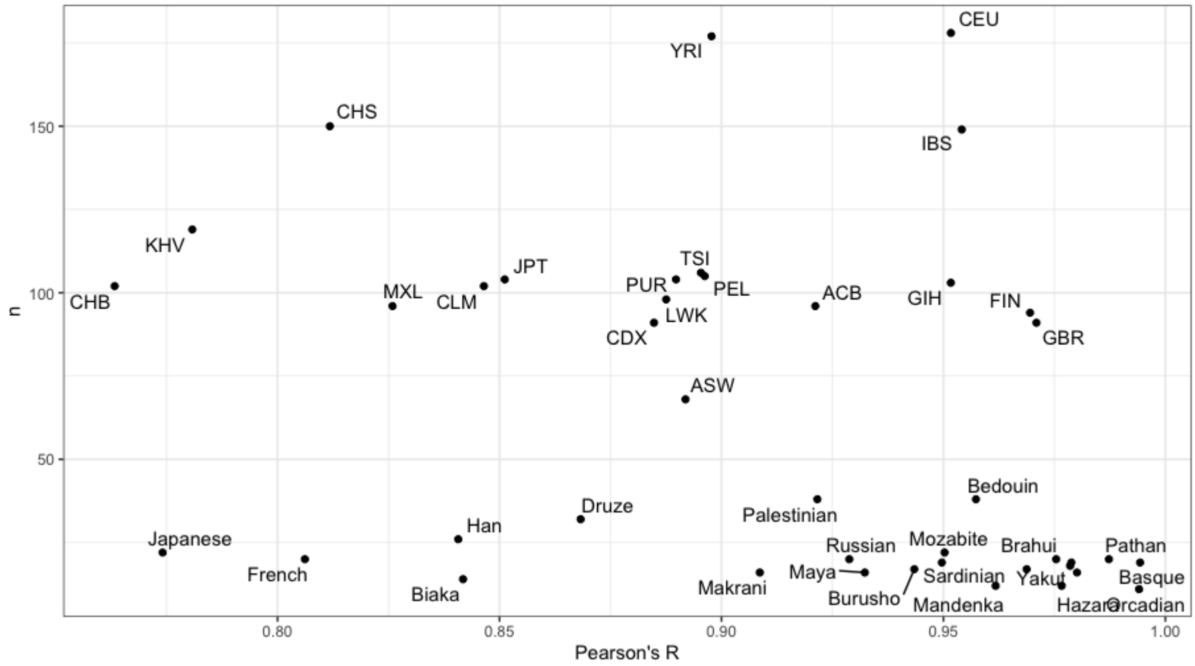

Web Table 3. Imputation performance within each population in HGDP and 1000G

Population	No. of Samples	Correlation	Concordance	Within 2 Repeat Lengths
HGDP				
Orcadian	11	0.99	0.45	1.00
Hazara	12	0.98	0.50	0.92
Mandenka	12	0.96	0.17	0.67
Biaka	14	0.84	0.21	0.36
Kalash	16	0.98	0.50	0.88
Makrani	16	0.91	0.50	0.88
Maya	16	0.93	0.62	0.94
Burusho	17	0.94	0.47	0.88
Yakut	17	0.97	0.41	0.94
Sindhi	18	0.98	0.39	0.89
Balochi	19	0.98	0.53	0.95
Basque	19	0.99	0.58	1.00
Sardinian	19	0.95	0.37	0.95
Brahui	20	0.98	0.65	0.90
French	20	0.81	0.55	0.80
Pathan	20	0.99	0.65	1.00
Russian	20	0.93	0.65	0.90
Japanese	22	0.77	0.23	0.82
Mozabite	22	0.95	0.50	0.86
Han	26	0.84	0.23	0.77
Druze	32	0.87	0.38	0.84
Bedouin	38	0.96	0.53	0.87
Palestinian	38	0.92	0.42	0.87
1000G				
ASW	68	0.89	0.37	0.72
CDX	91	0.88	0.36	0.81
GBR	91	0.97	0.67	0.93
FIN	94	0.97	0.74	0.91
ACB	96	0.92	0.32	0.68
MXL	96	0.83	0.55	0.91
LWK	98	0.89	0.19	0.66
CHB	102	0.76	0.38	0.86
CLM	102	0.85	0.54	0.93
GIH	103	0.95	0.31	0.87
JPT	104	0.85	0.30	0.76
PUR	104	0.89	0.46	0.82
PEL	105	0.90	0.69	0.96
TSI	106	0.90	0.57	0.86
KHV	119	0.78	0.29	0.78
NA	127	0.91	0.51	0.83
IBS	149	0.95	0.63	0.90
CHS	150	0.81	0.41	0.85
YRI	177	0.90	0.35	0.73
CEU	178	0.95	0.58	0.89

In summary, imputation performance was reasonable for identifying whether the total repeat length across both alleles was similar. Exact concordance was less likely, but the proportion of samples where the total repeat length was more than 2 repeats away was <20%.

Impact of filtering post genotype

As Beagle computes posterior probabilities for each called genotype, we perform some further testing to measure whether the computed posterior probability was useful in filtering low quality calls. As Beagle is generally used to call biallelic variants, it was not known whether the reported genotype posterior probability would have relevance in a multiallelic (64 separate STR length) setting.

To do this, we used the 1000G data to test whether the reported posterior probability at each genotype related to the probability of that genotype being correct. Web Figure 4A shows the histogram posterior probability for each reported genotype. The vast majority (79.3%) of reported posterior probabilities were one, with the next commonest reported probabilities being 0.99 (3.6%) and 0.98 (1.86%). Despite this, there was almost no correlation between the reported posterior probability and absolute mean difference in repeat length (Web Figure 4B). There was a slight correlation between the reported posterior probability and repeat length, with longer repeats more likely to have higher posterior probabilities (Web Figure 4C). For completeness, we then re-ran the analysis calculating repeat length based on the average repeat length summed across all posterior probabilities. i.e., for a sample that had an estimated repeat length of 48 (posterior probability 0.8) and 42 (posterior probability 0.2), we would calculate the repeat length as 48 * 0.8 + 42 * 0.2 = 46.8 This approach is shown in Web Figure 4D, making clear that the addition of this methodology makes little difference (as would be expected, with most genotypes being called with >95% reported probability).

Web Figure 4. Effect of filtration on accuracy of genotype calls: A) Histogram of reported posterior probabilities, B) Posterior probability plotted against absolute difference in repeats, C) posterior probability plotted against repeat length, and D) Comparison of taking the most probable allele vs the full posterior approach.

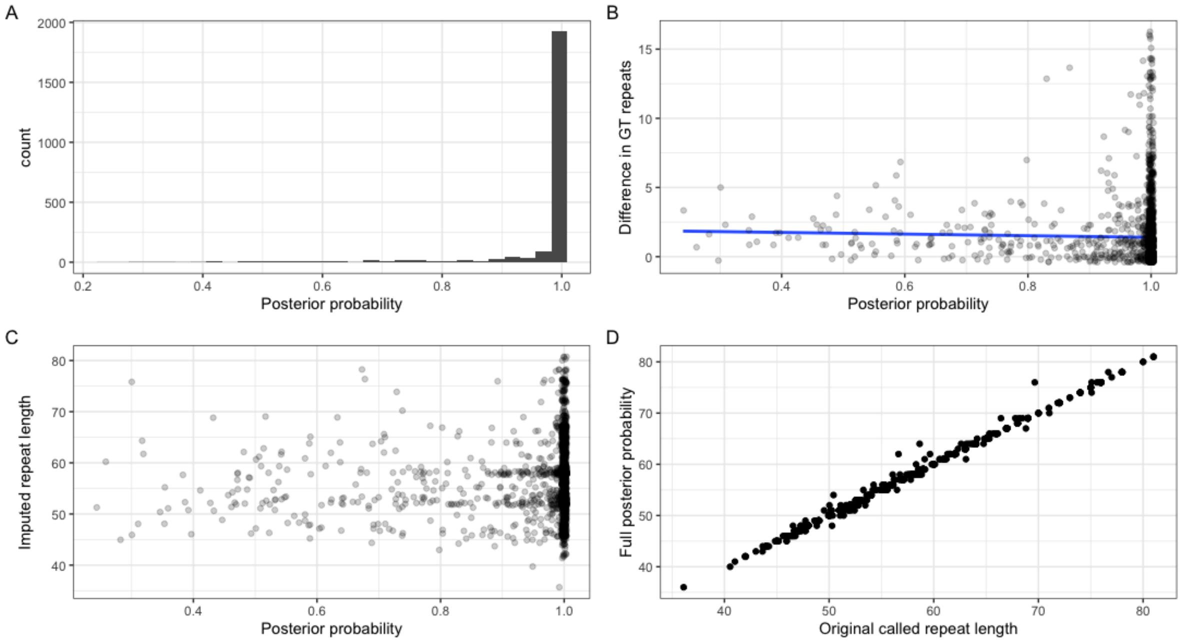

Web Table 4. ICD codes associated with each infection

Condition	ICD Codes
Sepsis	A02, A39, A40, A41
Endocarditis	I33, I38, I39
Pneumonia	J10,J12-J18, J86

Web Figure 5. Overall imputation performance at the *HMOX1* STR. The Y axis represents the true repeat length (summed number of GT(n) polymorphisms), called from whole genome sequencing, while the X axis represents the imputed repeat length from the gene array. Each dot represents an individual. Data from UK Biobank, 2006-onwards, UK.

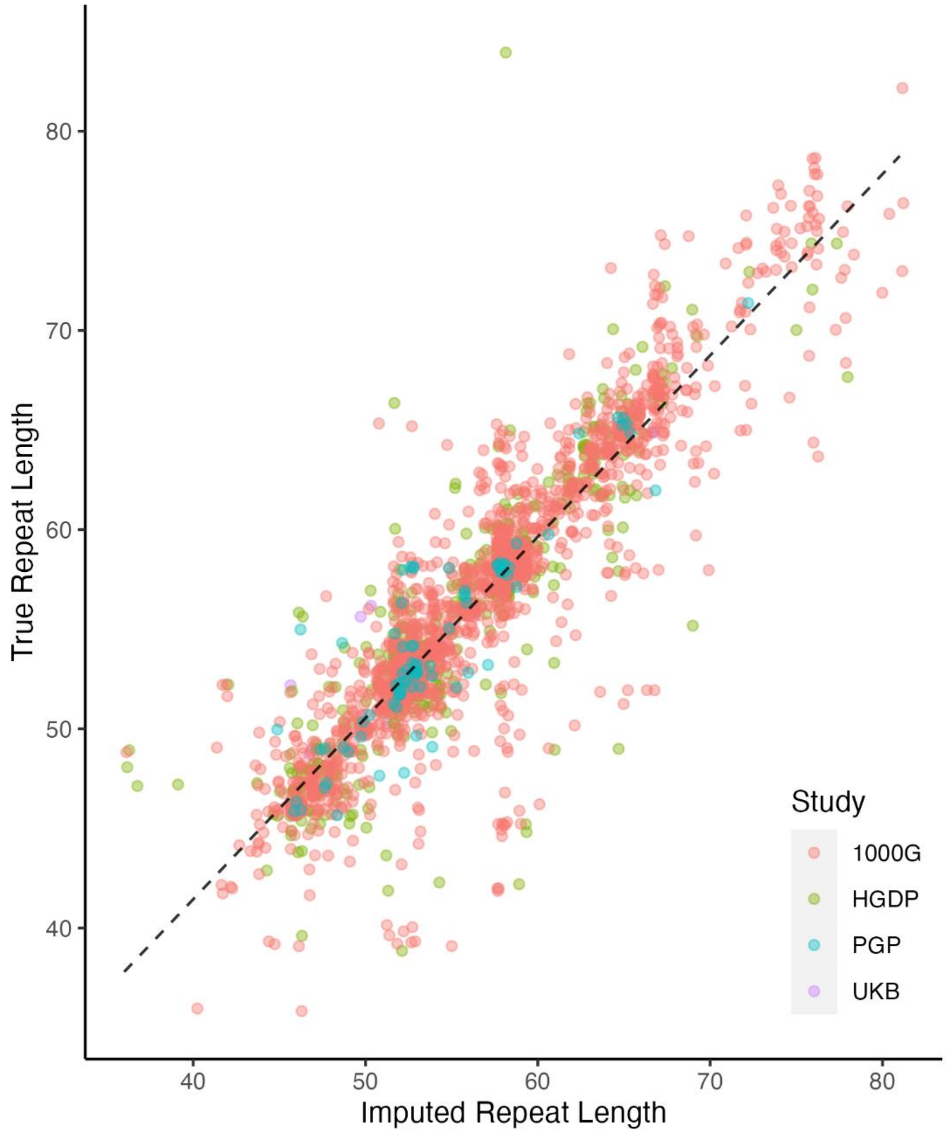

Web Figure 6. Absolute difference in repeat length and true repeat length across all four cohorts.

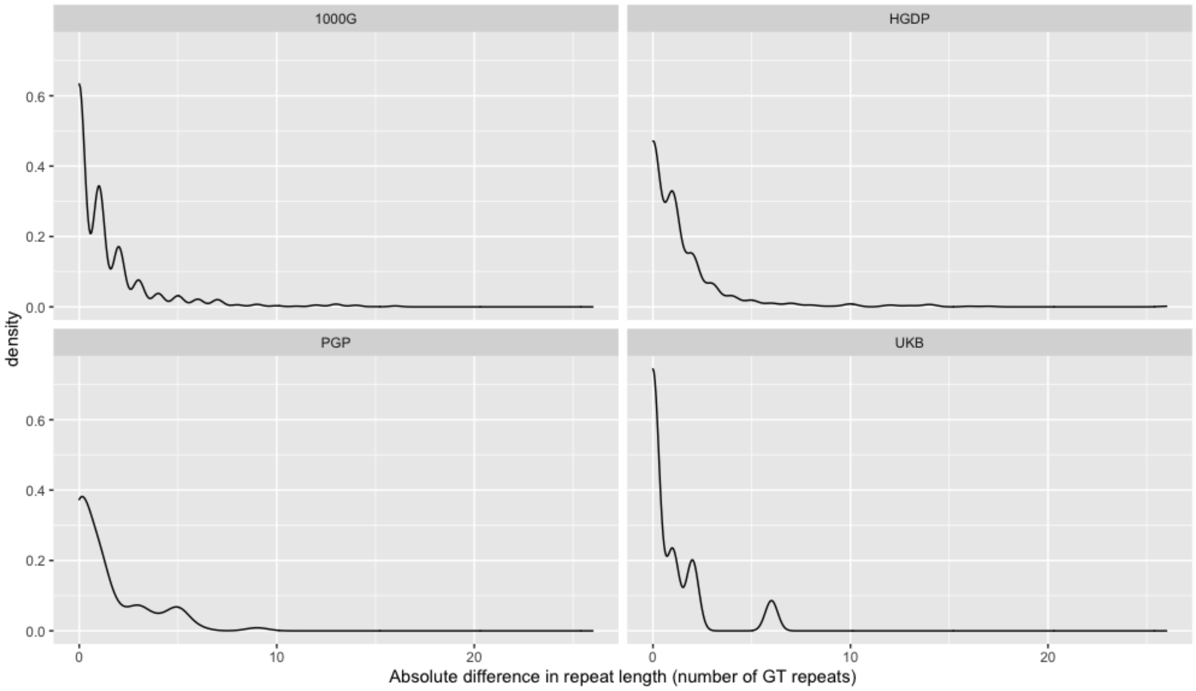

Web Figure 7. Change in height with increasing number of (AC)n repeats at *RFT1* (showing genotypes where n>500, mean, with bar representing standard error of the mean). Y axis represents standardised change in height, while X axis represents the number of repeats. Data from UK Biobank, 2006-onwards, UK.

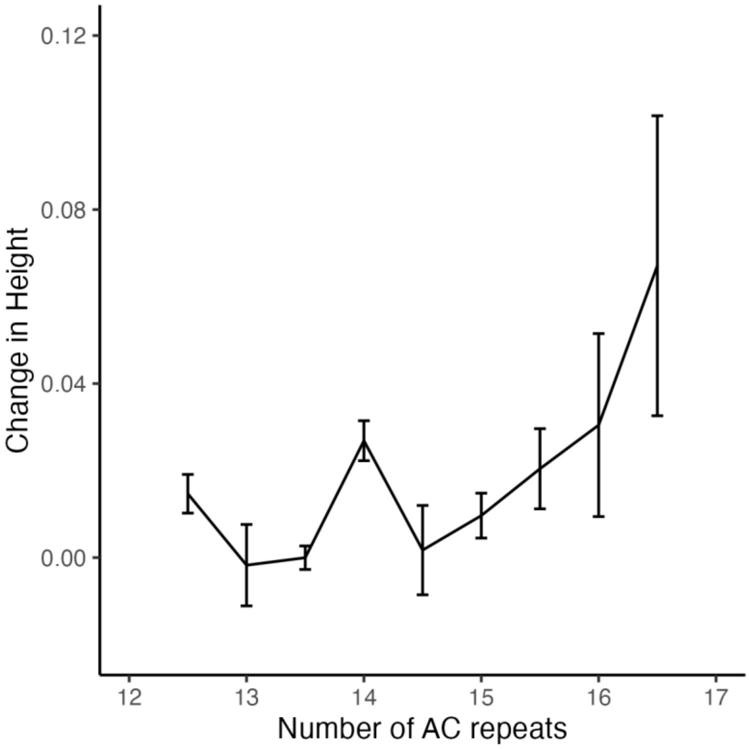

Web Figure 8. *HMOX1* Repeat lengths in UK Biobank (2006-onwards, UK) from a) individual alleles, and b) summed across both alleles for each individual. Y axis is on a log10 scale and represents the count of alleles.

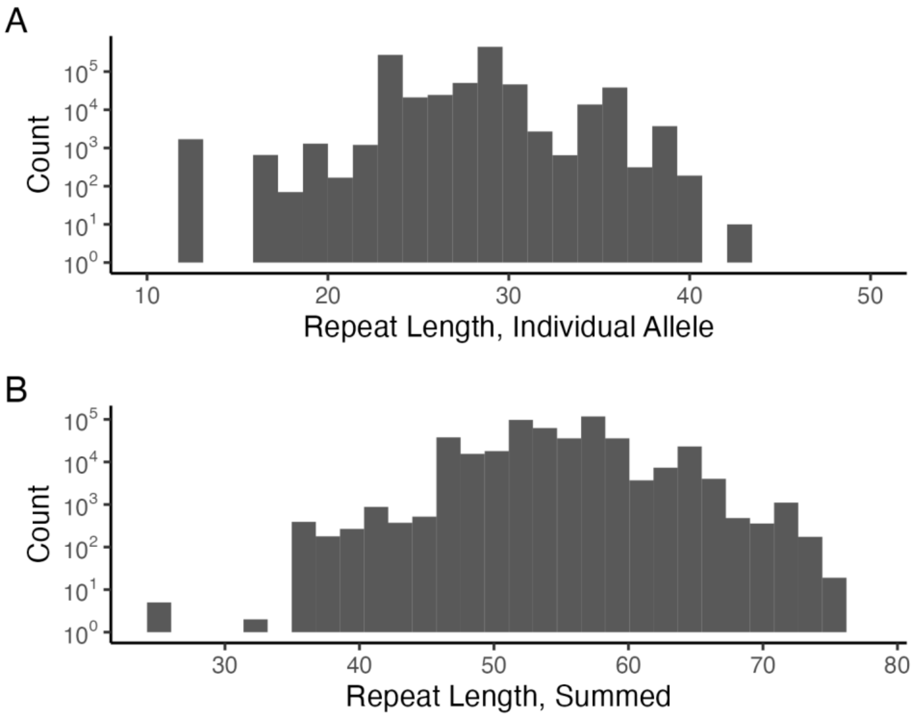

Web Figure 9. Quantile-Quantile plot of the p values from the estimates from the phenome wide association study of the *HMOX1* GT(n) repeat length on a number of clinical and demographic variables from UK Biobank, UK, 2006-onwards.

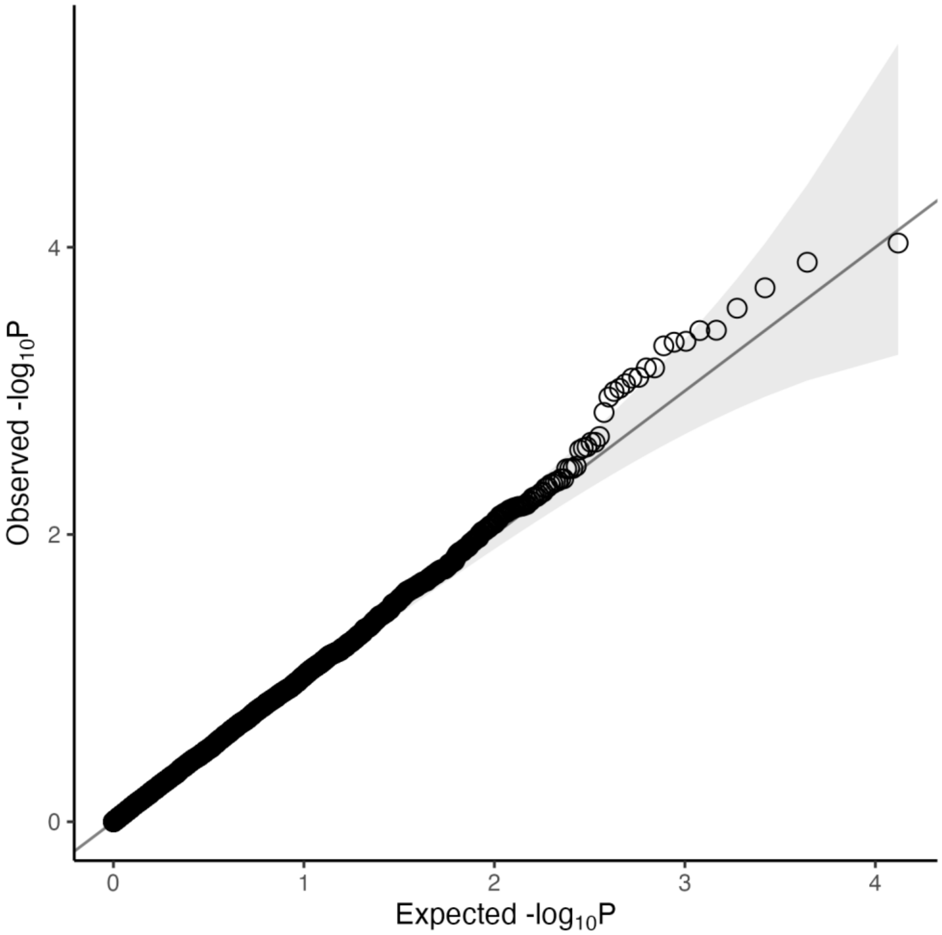

(See separate file for Web Figure 10.)

Web Figure 11. Comparison between measured bilirubin (y axis) and, the day of post-natal sampling, in a cohort of babies from the Avon Longitudinal Study of Parents and Children, UK, 1990-1991.

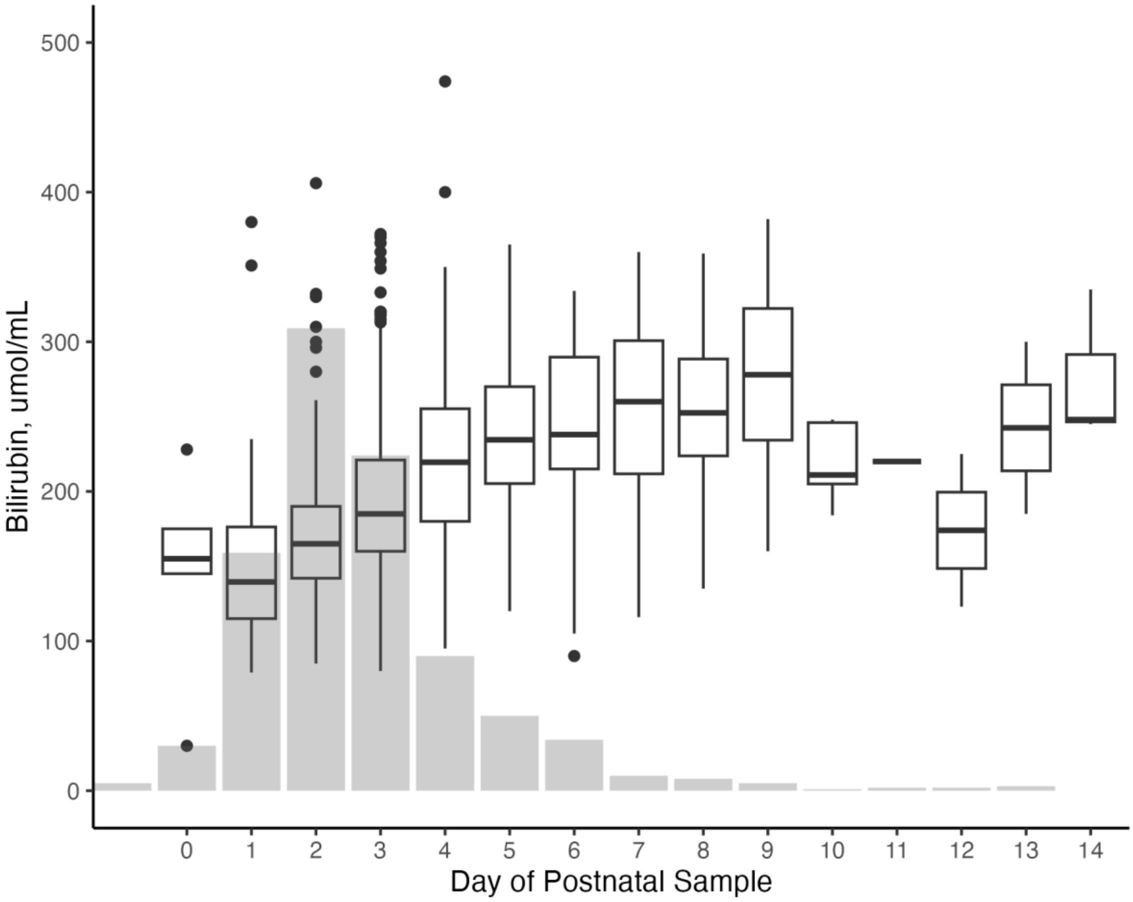

Web Figure 12. Association between bilirubin Z-score and summed *HMOX1* repeat length (number of GT(n) polymorphisms). Data from Avon Longitudinal Study of Parents and Children, collected 1990-1991, UK.

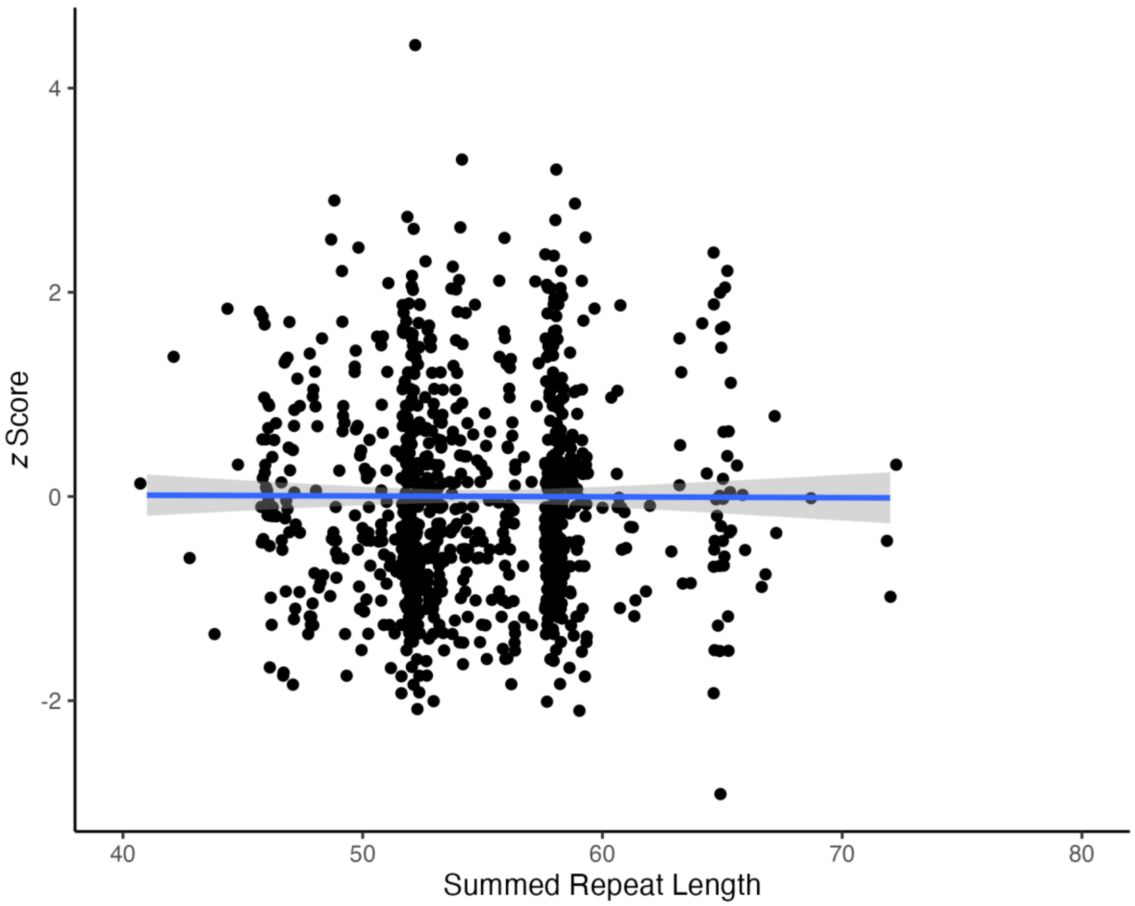

References

1. Saini S, Mitra I, Mousavi N, Fotsing SF, Gymrek M. A reference haplotype panel for genome-wide imputation of short tandem repeats. *Nat Commun*. 2018;9(1):4397. http://dx.doi.org/10.1038/s41467-018-06694-0

2. Cavalli-Sforza LL. The Human Genome Diversity Project: past, present and future. *Nat Rev Genet*. 2005;6(4):333–340. http://dx.doi.org/10.1038/nrg1596

3. Mitchell R, Hemani G, Dudding T, Corbin L, Harrison S, Paternoster L. UK Biobank Genetic Data: MRC-IEU Quality Control, version 2. 2019 [cited 2021 Dec 21]. https://data.bris.ac.uk/data/dataset/1ovaau5sxunp2cv8rcy88688v
